# Local ablation or radioembolization of colorectal cancer metastases: comorbidities or older age do not affect overall survival

**DOI:** 10.1186/s12885-018-4784-9

**Published:** 2018-09-10

**Authors:** Ricarda Seidensticker, Robert Damm, Julia Enge, Max Seidensticker, Konrad Mohnike, Maciej Pech, Peter Hass, Holger Amthauer, Jens Ricke

**Affiliations:** 1 Department of Radiology, Ludwig-Maximilians-University Munich, Munich, Germany; 2 Department of Radiology and Nuclear Medicine, Otto-von-Guericke-University Magdeburg, Leipziger Str. 44, 39120 Magdeburg, Germany; 3 Diagnostic and Treatment Center Frankfurter Tor, Berlin, Germany; 4 Department of Radiation Oncology, Otto-von-Guericke-University Magdeburg, Berlin, Germany; 5 Department of Nuclear Medicine, Charite, Berlin, Germany; 6 Deutsche Akademie für Mikrotherapie e.V, Magdeburg, Germany

**Keywords:** Colorectal cancer, Elderly patients, Comorbidities, Multimodal therapy

## Abstract

**Background:**

Local ablative techniques are emerging in patients with oligometastatic disease from colorectal carcinoma, commonly described as less invasive than surgical methods. This single arm cohort seeks to determine whether such methods are suitable in patients with comorbidities or higher age.

**Methods:**

Two hundred sixty-six patients received radiofrequency ablation (RFA), CT-guided high-dose rate brachytherapy (HDR-BT) or Y90-radioembolization (Y90-RE) during treatment of metastatic colorectal cancer (mCRC). This cohort comprised of patients with heterogenous disease stages from single liver lesions to multiple organ systems involvement commonly following multiple chemotherapy lines. Data was reviewed retrospectively for patient demographics, previous therapies, initial or disease stages at first intervention, comorbidities and mortality. Comorbidity was measured using the Charlson Comorbidity Index (CCI) and age-adjusted Charlson Index (CACI) excluding mCRC as the index disease. Kaplan-Meier survival analysis and Cox regression were used for statistical analysis.

**Results:**

Overall median survival of 266 patients was 14 months. Age ≥ 70 years did not influence survival after local therapies. Similarly, CCI or CACI did not affect the patients prognoses in multivariate analyses. Moderate or severe renal insufficiency (*n* = 12; *p* = 0.005) was the only single comorbidity identified to negatively affect the outcome after local therapy.

**Conclusion:**

Interventional procedures for mCRC may be performed safely even in elderly and comorbid patients. In severe renal insufficiency, the use of invasive techniques should be limited to selected cases.

## Background

Age is a major risk factor for colorectal cancer (CRC) and cancer in general [1]. Elderly patients often suffer from comorbidity and reduced organ function thus requiring particular considerations when making treatment decisions. Additionally elderly patients present a very heterogeneous group with chronological age being insufficient to describe individual resources and deficits. Contributing to these difficulties in decision making, elderly patients are underrepresented in cancer trials while they account for most of the actual patients [2]: When analyzing 495 NCI (National Cancer Institute) studies, Lewis et al. found that only 32% of cancer trial participants were age 65 years and older, in contrast to 61% in the US cancer population [3]. Other authors have published similar results, with an even greater difference for patients aged 70 years and older [4]. Although there is evidence that age should not be a reason to refrain from surgery and chemotherapy, most studies comprise a higher age and comorbidities as exclusion criteria [5–7]. In clinical practice, patients at higher age or with comorbidities often receive the recommended chemotherapies at reduced doses outside the standard prescription [8–10]. Yet, the effectiveness of such adapted therapy regimen is unknown.

Local ablative treatments (LAT, e.g. radiofrequency/microwave ablation and interstitial HDR-brachytherapy) as well as locoregional therapies (e.g. Y90 radioembolization) offer local tumor control and extensive cytoreduction with low morbidity and mortality. In oligometastatic disease with few tumor sites and limited number of metastases, LAT can achieve long-term disease control by complete tumor ablation in patients not eligible for surgery [11]. In contrast, locoregional therapies such as Y90 radioembolization may contribute to the overall survival of selected patient by improving the local response in liver-dominant disease or by providing a salvage treatment in chemo-refractory liver metastases [12, 13]. Accordingly, the toolbox of local ablative treatments and locoregional therapies was included in the latest ESMO guideline for colorectal cancer with oligometastatic disease or liver dominant, chemo-refractory metastases [14]. In the context of elderly and comorbid patients, data on the efficacy of LAT is still rare.

This study aims to assess the influence or absence of negative effects of higher age or comorbiditieson the outcome after local therapies. We hypothesize that minimal-invasive local or locoregional techniques add further value by offering broader treatment options in elderly and comorbid patients with metastatic colorectal disease.

## Methods

### Patient cohort

We searched our institutional data base for all patients with mCRC receiving at least one radiofrequency ablation (RFA), high-dose-rate brachytherapy (HDR-BT) or Y90-radioembolization (Y90-RE) between 2006 and 2010. We included all patients with complete records on patient history and at least one follow up visit.

The study comprised a total of 266 patients (179 male, 87 female; mean age 66 years). One hundred ninety-six patients (73.7%) had synchronous metastases within 12 months after diagnosis of the primary tumor. Nearly all patients presented with hepatic metastases (*n* = 251, 94.4%). Further sites of dissemination included lung (*n* = 77, 28.4%), lymphatic (*n* = 44, 16.5%), osseous (*n* = 10, 3.8%) or other metastases (*n* = 22, 8.3%). Most of the patients failed at least one (*n* = 79, 29.7%) or two (*n* = 160, 60.2%) lines of chemotherapy compromising either irinotecan or oxaliplatin combined with 5-fluorouracil. Additionally, 169 patients (63.5%) received EGFR or VEGF inhibiting therapy. Prior surgical treatments included surgery for the primary tumor in 263 patients (98.9%), resection of hepatic metastases in 91 patients (34.2%) and resection of lung/other metastases in 34 patients (12.8%).

Throughout the observation period, nearly half of the patients developed further liver metastases (*n* = 118, 44.4%) followed by lung metastases (*n* = 108, 40.6%), lymphatic metastases (*n* = 51, 19.2%), osseous metastases (*n* = 18, 6.8%) and other (*n* = 73, 27.4%).

Patients were considered for local ablative treatment and Y90 radioembolization by a multidisciplinary team (MDT; including medical, surgical and radiation oncologists) depending on their stage of disease (e.g. size of tumor, number of lesions, tumor sites) as well as organ function and performance status. Local ablation was selected in potentially resectable metastases only if patients had an unfavorable performance status and/or severe comorbidities (resulting in a high risk of perioperative morbidity and mortality) or if patients refused surgery. Patients with single lesions up to 3 cm in diameter were preferably treated by radiofrequency ablation. If the localization and number of metastases or tumor size above 3 cm limited RFA, interstitial HDR brachytherapy was applied for oligometastatic disease. Patients with diffuse, liver-dominant involvement underwent Y90 radioembolization. In case of tumor progress during follow-up, patients were reassessed by the MDT for the next treatment step, i.e. further local treatment strategies and/or systemic therapy. In total, 732 interventions were performed.

### Local and locoregional therapies

The following image guided techniques were considered by the MDT (if not eligible for systemic therapy only).

#### Radiofrequency ablation

Radiofrequency ablation induces a coagulation necrosis of tumor tissue by generating heat [15]. RFA is considered to be a safe and effective method with major complications occurring in 1–5% of patients. Beside limitations according to proximity to vulnerable organs, RFA underlies a heat-sink effect restricting the maximum size of the coagulation necrosis [16].

In our study, local ablation for smaller lung or liver metastases (< 3 cm) was performed using CT-guided radiofrequency ablation (LeVeen®, Boston Scientific, Natrick, United States or Starburst Semi-Flex®, AngioDynamics, Mountain View, Canada) according to manufacturer’s specifications. A total of 21 liver and 77 lung RFA interventions were conducted.

#### CT guided high-dose rate brachytherapy

CT-guided HDR-BT is an ablative technique utilizing radiation from an Iridium-192 source in afterloading technique. Interstitial catheters were inserted by CT-guidance and subsequent 3D treatment planning was applied (Oncentra®, Nucletron, Veenendaal, The Netherlands). As the catheters are fixed within the tumor, the delivery of irradiation is not affected by breathing motion. As a consequence, dose delivery to the tumor is highly accurate and exposure of healthy tissues or risk organs can be reduced to a minimum [17].

Since HDR-BT has no systematic restrictions for tumor size and location close to vessels, it was preferably indicated if multiple tumors were present as well as in larger (> 3 cm) liver or lung metastases or any lymphatic metastases [18–20]. To ensure a complete ablation, a target dose of 20Gy in a single session was subscribed [21]. HDR-BT was mainly used for liver ablations (*n* = 422), as well as for ablation of lung metastases (*n* = 52), lymphatic nodes (*n* = 9) and other tumor sites (*n* = 8).

#### Y90-radioembolization

If number, size or location of liver metastases exceeded the capabilities of local ablation by RFA or HDR-BT, patients were subsequently evaluated for loco-regional radioembolization using microspheres labeled with the beta-emitter Yttrium-90 (half-life 64 h; mean energy 0.96 MeV) administered through an angiographic catheter to the liver arteries [22, 23]. Multinodular liver metastases were treated in 96 cases by 142 radioembolizations using Y90 resin microspheres (SIR-Spheres®, Sirtex Medical, Lane Cove, Australia), the required dose was calculated previously according to the body-surface area method after an initial evaluation with Technecium-99 m macro-aggregated albumin (LyoMAA, Covidien, Neustadt, Germany).

### Comorbidity measurement

To assess comorbidities, we used the Charlson Comorbidity Index (CCI) which is validated in older patients with the option to calculate an age adjusted index (Charlson Age Comorbidity Index, CACI) [24, 25] to predict mortality in a range of comorbid conditions. 19 comorbidity items were included and each condition was assigned a score of 1, 2, 3 or 6 (see Table 3), depending on the risk of death associated with each one. The sum of these items (between 0 and 30) formed the final comorbidity index (CCI, CACI) that has been established as a predictor of patient outcome and mortality in different settings and larger populations including cancer patients [26]. The index disease, metastatic colorectal cancer, was excluded when calculating the index. Additional information was assessed regarding typical cardiovascular risk factors not included within the CCI (e.g. hypertension, hyperlipidemia, obesity).

All information on comorbidity was recorded at baseline.

### Statistical analysis

SPSS 21.0 (IBM®, New York, USA) was used for the complete analysis set. Comorbidity items including the summary within the CCI/CACI, patient age and key characteristics of disease and treatment underwent a stepwise Cox regression analysis. All baseline variables were initially analyzed in a univariate Cox regression. Any variable scoring a *p*-value < 0.1 was then included in a multivariate Cox proportional hazard model. Tables 3 and 4 give a summary of the main analysis with *p*-values, harzard ratios (HR) and 95% confidence intervals (95% CI). Statistical significance in the multivariate analysis was assumed for *p*-values < 0.05. Visualization was achieved by Kaplan-Meier charts.

## Results

### Treatment outcome

A total of 732 procedures were performed in all patients, an overview is given in Table 1. All survival data were measured beginning with the first treatment at our institution.

**Table 1:** Overview on procedures and outcome

			Median overall survival (months)^a^			
	Patients	Procedures	Patient age		Comorbidity	
	*n*	*n*	≥ 70 years	< 70 years	CCI ≥ 3	CCI < 3
RFA	60	99	26.7 m	24.3 m	24.0 m	26.2 m
liver	18	21		**(*p*** **= 0.76)**		**(*p*** **= 0.16)**
lung	42	77				
other	1	1				
HDR-BT	192	491	19.1 m	18.2 m	16.4 m	18.9 m
liver	176	422		**(*p*** **= 0.83)**		**(*p*** **= 0.43)**
lung	29	52				
lymph node	9	9				
other	8	8				
Y90-RE	96	142	6.9 m	6.5 m	5.3 m	6.9 m
				**(*p*** **= 0.86)**		**(*p*** **= 0.21)**

^a^statistics for overall survival according to Cox regression analysis; *p*-values (bold) refer to the comparison of survival between age/comorbidity groups

#### RFA patients

Patients initially presenting with singular, small metastases (< 3 cm) confined to lung (*n* = 42) or liver (*n* = 18) were treated by radiofrequency ablation yielding a median survival of 26.7 months and 24.4 months (including further local ablative treatments and/or systemic therapies in case of disease progression). A single RFA treatment was used for the ablation of a vertebral metastasis. 50 out of 60 patients (83%) treated by RFA underwent multiple RFA sessions and/or further treatment by HDR-BT for recurrent metastases.

#### CT-guided HDR brachytherapy patients

Oligonodular and larger metastases were treated by HDR-BT. Patients with liver metastases eligible for HDR-BT at their first presentation in our department achieved a median survival of 18.1 months (*n* = 176). Initially applying HDR-BT to lung metastases, a median survival of 29.6 months was observed (*n* = 29). Lymphatic nodes and other infrequent localizations of metastases (e.g. adrenal glands, pancreas) were treated exclusively by HDR-BT with a corresponding median survival of 17.0 to 26.7 months. In patients with multiple tumor sites or disease progression during follow up, HDR-BT was repeated (*n* = 143) or Y90-RE performed (*n* = 28).

#### Radioembolization patients

Ninety- six patients with diffuse liver metastases underwent Y90-RE with a median survival of 6.7 months. 68 of these patients who had failed first and second line chemotherapy including variable treatment cycles with oxaliplatin, irinotecan and 5-fluorouracil demonstrated a significantly shorter median survival of 5.8 months in univariate and multivariate Cox regression analyses (*p* < 0.001)*.* However, 19 salvage patients (28%) undergoing Y90 radioembolization had a survival of at least 9 months with long-term survivors reaching a survival of nearly 30 months. All salvage patients treated by Y90-RE in this cohort represent a majority of patients in a dedicated prognostic analysis which can be reviewed for supplementary information [27].

### Impact of palliative chemotherapy after first interventional treatment

A total of 120 patients (45%) received further chemotherapy after the first local treatment. These patients demonstrated an improved survival of 22.0 vs. 16.1 months compared to patients without further systemic therapies (*p* = 0.009; HR 0.71; 95% CI 0.55–0.92).

### Outcome by patient characteristics

Overall patient characteristics are outlined in Table 2. Survival in all patients accounted for 14 months, survival analysis was conducted using a stepwise Cox regression analysis. Nearly all patients suffered from liver metastases (*n* = 251). Patients with an initial positive N stage (*n* = 179) and metachronous lymph node metastases (*n* = 44) had a poorer prognosis (13.1 vs 17.0 months; 9.8 vs 16.1 months) after first interventional treatment in univariate analysis, yet multivariate regression analysis did not demonstrate a significant influence on overall survival (*p* = 0.25 and *p* = 0.17; respectively).

**Table 2:** Patient characteristics

	*n*	%
All patients	266	100.00
Male	179	67.30
Female	87	32.70
First diagnosis		
Mean age (SD) in years	63.0 (+/− 9.7)	
Primary tumor		
located in Colon (C18)	151	56.77
Rectosigmoid junction (C19)	18	6.77
Rectum (C20)	98	36.84
T1,T2	29	10.90
T3,T4	227	85.30
T missing	10	3.80
N0	74	27.80
N1,2	179	67.30
N status missing	13	4.90
Synchronous metastases	166	62.41
Prior treatment		
Systemic chemotherapy	248	93.23
Median lines of chemotherapy (range)	2 (0–8)	
Radiochemotherapy	30	11.28
Surgery for colorectal primary	263	98.87
Radiation therapy for colorectal primary	21	7.89
Surgery for liver metastases	91	34.21
Other local treatment for liver metastases	40	15.04
Surgery for lung metastases	14	5.26
Surgery for other metastases	20	7.52
Local therapy for other metastases	5	1.88
First interventional treatment		
Mean age (SD) in years	66,5 (+/−9.6)	
Age > 70 years	89	33.5
Median Karnofsky index (range) in %	80 (50–100)	
Liver metastases	251	96.99
Liver metastases only	121	45.50
Liver involvement > 25%	45	16.92
Lung involvement	100	37.60
Other	83	31.20
≥ 2 organ systems involved	140	52.63

Synchronous metastases at first diagnosis (*n* = 166) only had significant influence in univariate analysis (*p* = 0.036) but not in multivariate analysis (*p* = 0.90). Metachronous pulmonary metastases had no impact on survival (*p* = 0.55).

Systemic therapy options after initiation of interventional therapy were stratified by previous failure of either oxaliplatin or irinotecan based combined regimen (second line, *n* = 79) or failure of both (third line, *n* = 160). Patients without prior chemotherapy were classified to first line (*n* = 27), including patients with contraindications to systemic therapy. A median survival of 13.2 vs. 16.6 months was observed in patients receiving third line therapy compared to patients in earlier lines of therapy without prognostic influence in multivariate analysis (*p* = 0.30).

If third line patients were still eligible for local-ablative techniques (RFA and/or HDR-BT), the median survival reached 17.5 months (*n* = 114).

The complete multivariate analysis is demonstrated in Table 4.

### Age analysis

**Fig. 1:**
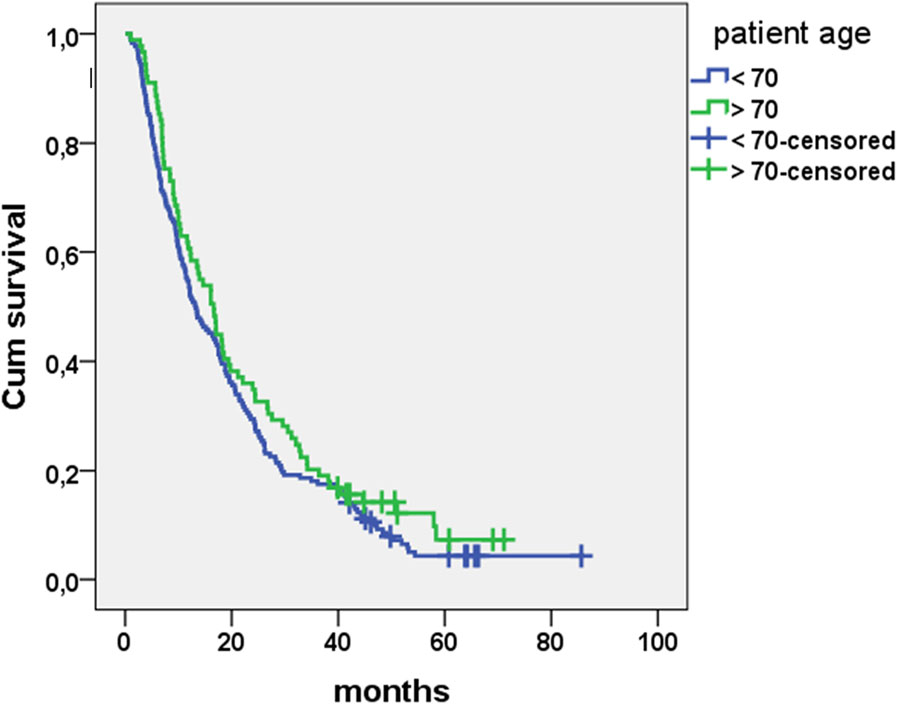
Overall survival by age. Kaplan Meier estimation for overall survival after first treatment by age <  70 (13.2 months; *n* = 177) and age ≥ 70 (16.6 months; *n* = 89), no statistical difference between groups (*p* = 0.19; Cox regression analysis)

Our cohort included 89 patients (33.5%) 70 years or older. This patient group demonstrated no altered survival as compared to younger patients after first interventional therapy in a Cox regression analysis (*p* = 0.19; HR 0.84; 95% CI 0.64–1.10). Median survival in the subgroup of elder patients was 16.6 vs. 13.2 months as shown in Fig. 1. In patients older than 70 years initial or additional lymphatic metastases were of no prognostic value (*p* = 0.11; HR 1.24; 95% CI 0.95–1.61 and *p* = 0.23; HR 1.62; 95% CI 0.74–3.54), just as for heavily pretreated patients with at least three lines of systemic chemotherapy (*p* = 0.18; HR 1.23; 95% CI 0.91–1.67). Survival of elderly versus younger patients was similar regarding the first technique applied (RFA, HDR-BT or Y90-RE) in regression analysis, see Table 1.

### Comorbidity analysis (CCI, CACI)

With a sum of 3 points or more for the CCI, 43 patients (16.2%) displayed severe comorbidities at baseline. These comorbidities were significantly more frequent in older patients ≥70 years (*n* = 21; 23.6%) than in younger patients < 70 years (*n* = 22; 12.4%; *p* = 0.023; Chi-Square test). According to the age adjusted CACI, a total of 112 patients (42.1%) were considered with severe comorbidities at first therapy. An overview of CCI/CACI in the patient cohort is given in Table 3.

**Table 3:** Prevalence of comorbidities according to the Charlson Comorbidity index

CCI^a^	condition	*n*	%	*p*-value°	HR (95% CI)
1	myocardial infarction, coronary artery disease	36	13,5	0.38	0.85 (0.58–1.23)
	congestive heart failure	15	5,6	0.62	0.87 (0.51–1.50)
	peripheral vascular disease	55	20,7	0.81	0.96 (0.71–1.31)
	cerebrovascular disease	13	4,9	0.58	0.85 (0.48–1.52)
	dementia	0	0		
	chronic pulmonary disease	21	7,9	0.046	0.61 (0.38–0.99)
	connective tissue disorder	2	0,8	0.93	0.94 (0.23–3.78)
	peptic ulcer disease	2	0,8	0.006	7.40 (1.80–30.50)
	mild liver disease	10	3,8	0.26	1.44 (0.76–2.72)
	diabetes without complications	42	15,8	0.94	0.99 (0.70–1.40)
2	diabetes with end-organ damage	17	6,4	0.74	0.92 (0.55–1.52)
	hemiplegia	1	0,4	0.70	1.48 (0.21–10.61)
	moderate/severe renal disease	12	4,5	0.005	2.3 (1.29–4.13)
	any tumor without metastases (incl. Leukemia, lymphoma)	34	12,8	0.89	1.03 (0.71–1.50)
3	moderate/severe liver disease	3	1,1	0.33	1.77 (0.57–5.55)
6	metastatic solid tumor (mCRC excluded)	0	0		
	AIDS	0	0		
8	AIDS and any tumor	0	0		

^a^age adjusted index CACI adds 1 point for each decade after 40 years

°statistics for overall survival according to univariate Cox regression, variables with univariate p < 0.1 are processed in Table 4

**Fig. 2:**
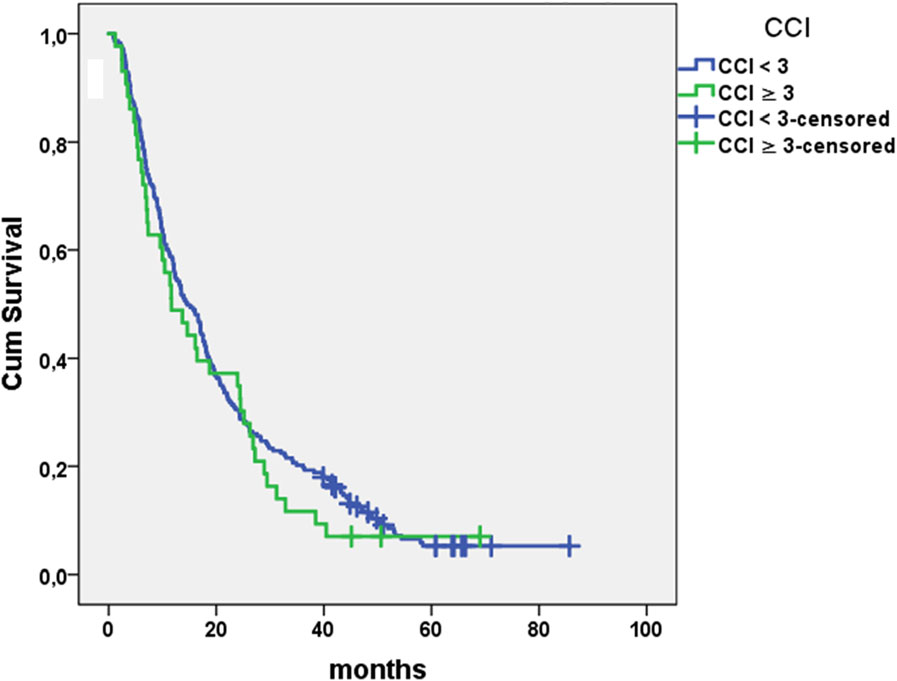
Overall survival by CCI. Kaplan Meier estimation for overall survival after first treatment separated by Charlson Comorbidity Index < 3 (21.9 months; *n* = 223) and ≥ 3 (18.1 months; *n* = 43); no statistical difference between groups (*p* = 0.41; Cox regression analysis)

In a univariate Cox regression, both CCI or CACI ranging from 0 to 7 and 0–8 had no significant impact on the patients prognosis (*p* = 0.82; *p* = 0.86), respectively. Comparison of patients with severe comorbidities (CCI ≥ 3) versus no or moderate comorbidities demonstrated no significant influence on overall survival either (18.8 months vs. 21.9 months; *p* = 0.41; see Fig. 2). Regression analysis of all single items summarized in the index (see Table 3) revealed a significant influence of moderate or severe renal disease in 12 patients (*p* = 0.005). Two patients with gastric or duodenal ulcer died after 3.7 and 5.7 months, respectively (*p* = 0.006). Patients with chronic pulmonary disease (*n* = 29) had a lower hazard ratio (*p* = 0.006; HR 0.61; 95 CI 0.38–0.99). No other comorbidity item had a considerable impact, despite 55 patients suffering from peripheral vascular disease and 36 patients with a history of myocardial infarction or coronary heart disease (*p* = 0.81 and *p* = 0.38). Multivariate regression analysis finally confirmed a statistical significant impact of moderate or severe renal disease in all patients (*p* = 0.005).

Apart from the conditions reflected in the CCI, 116 patients had been diagnosed with hypertension (43.6%), 18 patients with obesity (6.8%) and 20 with hyperlipidemia (7.5%). None of these factors demonstrated a significant influence on survival as demonstrated in Table 4.

**Table 4:** Stepwise Cox regression analysis of key characteristics at baseline including CCI items with univariate *p* < 0.1 (all items of CCI are shown in Table 3)

Variable	Univariate *p*	HR (95% CI)	Multivariate p*	HR (95% CI)
CCI items p < 0.1				
Chronic pulmonary disease	0.046*	0.61 (0.38–0.99)	0.30	0.76 (0.45–1.28)
Peptic ulcer disease	0.006*	7.40 (1.80–30.50)	0.17	2.75 (0.65–11.69)
Moderate/severe renal disease	0.005*	2.3 (1.29–4.13)	0.005	2.46 (1.32–4.57)
Comorbidities not included in CCI				
Hypertension	0.54	0.93 (0.72–1.20)		
Obesity	0.32	0.77 (0.47–1.28)		
Hyperlipidemia	0.48	0.84 (0.51–1.37)		
Patient and treatment characteristics				
Age > 70 years	0.19	0.84 (0.64–1.10)		
CCI ≥ 3	0.41	1.15 (0.82–1.62)		
Positive N stage of primary	0.004*	1.27 (1.08–1.50)	0.25	1.11 (0.93–1.33)
Synchronous metastases	0.036*	1.36 (1.02–1.81)	0.90	0.98 (0.71–1.35)
Metachronous lymph node metastases	0.032*	1.44 (1.03–2.00)	0.17	1.31 (0.89–1.91)
Metachronous pulmonary metastases	0.55	1.09 (0.83–1.43)		
1st/2nd Line vs. 3rd Line treatment	0.001*	0.83 (0.77–0.90)	0.30	0.89 (0.72–1.1)
Salvage treatment in Y90-RE	0.001*	2.17 (1.37–3.45)	< 0.001	4.35 (3.06–6.17)

*multivariate Cox regression analysis including all variables *p* < 0.1 in univariate analysis

## Discussion

### Interventional oncology in elderly patients

Metastatic colorectal cancer continues to be a major therapeutic challenge especially in elderly patients as prevalence of comorbidity is considered to be more frequent compared to the background population [28]. The corresponding interaction between cancer and comorbidity, and whether comorbidity leads to cancer diagnosis in earlier or later stages, is still object to ongoing discussions [29]. Furthermore, elderly and multimorbid patients are often not eligible for surgery or efficacious polychemotherapies [30].

In our group of metastatic CRC patients, about 62% had comorbidities according to the CCI. Adding conditions as hypertension, hyperlipidemia and obesity, 71% of patients were suffering from comorbidities, which is far more frequent than in other studies applying the CCI reporting a prevalence between 32 and 41% in metastatic or non-metastatic CRC patients [31].

Median survival after RFA as first local treatment of liver metastases was 24.4 months in our patients, which is consistent with existing data ranging from 24 to 36 months [32].

As HDR-BT is usually applied in metastases exceeding the technical feasibility of RFA in size and number, thus adding an unfavorable prognosis bias, a corresponding median survival of 18.1 months was found in those patients. A retrospective analysis by Collettini et al. demonstrated a comparable median survival of 18 months after HDR-BT of colorectal liver metastases [33].

Most patients undergoing Y90-radioembolization had previously failed all accessible chemotherapies leading to a median survival of 5.8 months in this group. However, one quarter of these patients survived 9 or more months including a small group of long term survivors > 2 years indicating that patient selection is of utmost importance in a salvage population [34]. This could be shown by our group in a previous study regarding the prognostic value of Karnofsky index, tumor load and tumor markers in patients undergoing Y90-radioembolization to help selecting appropriate patients [27].

When applying CCI and CACI to measure the prognostic impact of comorbidities in our patients, we did not observe a relation of higher index values with overall survival. It should be noted that about 42% of all patients had severe comobidities according to the age-adjusted index (CACI≥3). This finding supports the assumption that local ablative therapies such as RFA or HDR-BT, or a locoregional treatment such as Y90 radioembolization, can be safely applied in risk patients with a moderate toxicity profile or adverse event rate, respectively.

A similar relationship was seen recently by Jehn et al. in patients undergoing systemic therapy for mCRC as CCI and age showed no influence on survival [35]. In this population, adverse events were not found to be more frequent in elderly patients, although a significantly higher CCI was observed. Also response rates and survival were balanced irrespective of age and comorbidity. Further studies even discuss inferior outcome in younger patients, most probably caused by more aggressive tumor biology as compared to elder patients [36, 37]. With regard to our patients treated by local therapies, we observed a similar trend potentially related to a more favorable tumor biology in the eldery.

### Implications

Our study has demonstrated that older age or a higher rate of comorbidities with age (CCI and CACI) do not influence survival in metastatic colorectal cancer when patients are selected for local or loco-regional ablation by RFA, HDR-BT or Y90 radioembolization. A poorer survival was only seen in patients with moderate or severe renal impairment in our multivariate analysis. Renal disease in general is associated with a poor prognosis and has been reported to have a specifically negative impact on survival in different cancer populations [38].

### Limitations

A possible source of error in our analysis may result from data being derived from discharge diagnoses or follow up documentation in our own medical hospital records. Conditions treated by the general practitioner or subsequently in other hospitals may not have been completely represented in our data as a result of the studies retrospective nature. Furthermore, our sample is not necessarily representative for all mCRC patients with a comparatively high frequency of comorbidities in our cohort as compared to other studies. However, we hypothesize that these finding exclude a positive selection in our cohort.

## Conclusion

The tool box of image guided treatments proved to be safe and applicable even in patients of higher age or patients presenting with comorbidities. Our study results support offering ablative treatments to metastatic colorectal cancer patients even at advanced age or high Charlson indices.

## Abbreviations

CACI: Charlson Age Comorbidity Index
CCI: Charlson Comorbidity Index
CT: Computed tomography
ESMO: European Society for Medical Oncology
HDR-BT: High-dose rate brachytherapy
mCRC: metastatic colorectal cancer
MRI: magnetic resonance imaging
RFA: Radiofrequency ablation
Y90-RE: Y90-radioembolization
